# Clinicopathological Significance of Loss of ARID1A Immunoreactivity in Ovarian Clear Cell Carcinoma

**DOI:** 10.3390/ijms11125120

**Published:** 2010-12-13

**Authors:** Daichi Maeda, Tsui-Lien Mao, Masashi Fukayama, Shunsuke Nakagawa, Tetsu Yano, Yuji Taketani, Ie-Ming Shih

**Affiliations:** 1 Department of Pathology, Graduate School of Medicine, the University of Tokyo, Tokyo, Japan; E-Mails: daichimaeda@gmail.com (D.M.); mfukayama-tky@umin.net (M.F.); 2 Department of Pathology, National Taiwan University Hospital, and College of Medicine, Taipei, Taiwan; E-Mail: tlmao@ntu.edu.tw; 3 Department of Obstetrics and Gynecology, the University of Tokyo, Tokyo, Japan; E-Mails: nakagawas-tky@umin.ac.jp (S.N.); tetu-tky@umin.ac.jp (T.Y.); taketani-tky@umin.ac.jp (Y.T.); 4 Department of Pathology, Johns Hopkins Medical Institutions, Baltimore, Maryland, USA

**Keywords:** ovarian, ARID1A, pathology

## Abstract

Recent genome-wide analysis has demonstrated that somatic mutations in *ARID1A* (*BAF250*) are the most common molecular genetic changes in ovarian clear cell carcinoma (OCCC). *ARID1A* mutations, which occur in approximately half of OCCC cases, lead to deletion of the encoded protein and inactivation of the putative tumor suppressor. In this study, we determined the significance of loss of ARID1A immunoreactivity with respect to several clinicopathological features in a total of 149 OCCCs. First, we demonstrated that ARID1A immunohistochemistry showed concordance with the mutational status in 91% of cases with 100% sensitivity and 66% specificity. Specifically, among 12 OCCC cases for which ARIDA mutational status was known, ARIDIA immunoreactivity was undetectable in all 9 cases harboring *ARID1A* mutations and was undetectable in one of 3 cases with wild-type *ARID1A*. With respect to the entire cohort, ARID1A immunoreactivity was undetectable in 88 (59%) of 149 OCCCs. There was no significant difference between ARID1A negative and positive cases in terms of histopathologic features, age, clinical stage, or overall survival. In conclusion, this study provides further evidence that mutations in *ARID1A* resulted in loss of ARID1A protein expression in OCCC, although no significant differences between ARID1A positive and negative cases were observed with respect to any clinicopathological features examined.

## 1. Introduction

Recent genome-wide sequencing analyses of all exons and transcriptome in ovarian clear cell carcinomas (OCCC) have identified somatic mutations of *ARID1A* (the *AT-rich interactive domain 1A*), also known as *BAF250*, in approximately half of OCCC cases [1,2]. *ARID1A* is located within chromosome 1p36, a region frequently deleted in a variety of human neoplastic diseases including OCCC [3]. *ARID1A* encodes a large nuclear protein that interacts with several other proteins including the core ATPase, either BRG or BRM, to form a SWI/SNF chromatin remodeling complex [4,5]. While BRG or BRM is directly responsible for moving the SWI/SNF complex along the DNA strands in an ATP-dependent process, it is the non-catalytic subunit, in this case ARID1A, that has the ability to modulate target specificity and ATPase activity. It has been demonstrated that the chromatin remodeling activity of SWI/SNF plays an essential role in regulating gene expression [6] and is important in development and cellular differentiation as well as in tumor suppression [5,7,8]. Indeed, the frequent somatic mutations in *ARID1A* in OCCC suggest a major role for *ARID1A* in the pathogenesis of OCCC. The majority of *ARID1A* mutations in OCCC belong to either insertion or deletion of base pairs, leading to frame shifts and ending in stop codons. As a result, *ARID1A* mutations typically generate truncated proteins that are highly prone to degradation (Guan, unpublished result), a characteristic feature of classical tumor suppressors.

Inactivating mutations in tumor suppressors could participate, not only in tumor initiation, but also in tumor progression and response to therapy. In the current study, we asked whether loss of ARID1A protein expression had clinical significance in patients with OCCCs and whether loss of ARID1A correlated with any histopathological features in those cases. We first correlated the *ARID1A* mutation status and loss of ARID1A expression in selected cases to demonstrate the sensitivity and specificity of applying ARID1A immunoreactivity as a surrogate marker for *ARID1A* mutations. We then performed immunohistochemistry to assess ARID1A expression patterns on paraffin sections from a total of 149 cases of ovarian clear cell carcinoma with well annotated clinical follow up information. The findings from this report provide further evidence to characterize the biological and clinical significance of loss of ARID1A expression in OCCC.

## 2. Results and Discussion

### 2.1. Results

**Figure 1.**
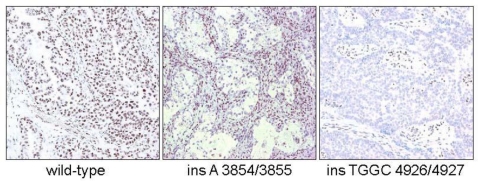
ARID immunoreactivity in three representative OCCCs which differ with respect to ARID1A mutational status. The OCCC with wild-type ARID1A shows intense and diffuse nuclear immunoreactivity in both tumor and stromal cells. In contrast, the other two cases, both with insertion mutations, exhibit undetectable ARID1A immunoreactivity while the stromal cells are positive, serving as internal positive controls.

We employed immunohistochemistry to evaluate the expression of ARID1A in OCCC tissues. Results of ARID1A immunohistochemistry in OCCCs are summarized in Table 1. ARID1A immunoreactivity was detected exclusively in nuclei of cells, and when protein expression was observed, it was always seen in a diffuse pattern. Positive immunoreactivity of ARID1A was recorded in 61 (41%) of 149 cases. Specifically, 88 (59%), 36 (24%), and 25 (17%) of 149 cases had a staining intensity score of 0, 1+, and 2+, respectively. Histological features in representative cases with different ARID1A immunostaining intensities and their mutational status are shown in Figure 1. Intra-tumoral non-neoplastic mesenchymal cells were usually strongly positive for ARID1A, and they served as positive controls, especially for negative cases.

In order to determine the biological significance of ARID1A expression in OCCC, we also analyzed ARID1A immunoreactivity in normal endometrium and adnexal endometriosis because OCCC most likely arises from endometrial glandular epithelium (Table 2). The results are shown in Table 2. All adnexal endometriosis cases (5/5) showed diffuse ARID1A immunoreactivity. Two cases showed 2+ positivity and 3 cases showed 1+ positivity. Normal endometrial glands also expressed ARID1A in all cases (38/38) regardless of menstrual phase. As compared to normal endometrium and endometriosis, OCCC showed a significantly higher frequency of a staining score equal to 0 (p < 0.0001, Fisher’s exact test). Therefore, we defined a staining score of 0 as a loss of ARID1A expression.

To determine if loss of ARID1A expression was associated with inactivating mutations of *ARID1A*, we correlated mutational status and immunoreactivity in 12 representative OCCCs. All OCCCs that harbored *ARID1A* mutations exhibited negative immunoreactivity, suggesting that *ARID1A* mutation resulted in loss of protein expression (Table 3). Among wild-type OCCCs (no *ARID1A* mutations), 2 of 3 cases exhibited diffuse ARID1A immunoreactivity; however, one case of OCCC with no *ARID1A* mutations demonstrated loss of ARID1A protein expression (score = 0).

**Figure 2.**
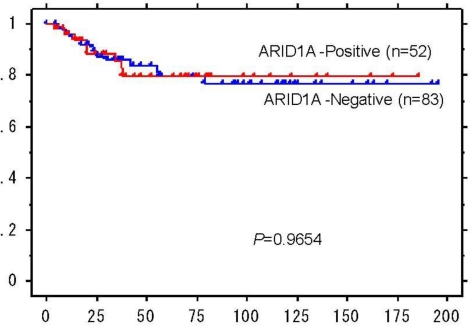
Kaplan-Meier analysis demonstrates lack of significance between ARID1A immunoreactivity and overall survival in multivariate analysis.

Next, we assessed the potential effects of loss of ARID1A expression on clinicopathological features of OCCCs by correlating ARID1A expression with several clinical and pathological characteristics of OCCC (Table 4). We found that there was no significant association of ARID1A expression and age at disease presentation, clinical stage, metastasis in lymph node, or histological features (p > 0.05). ARID1A immunoreactivity was not significantly correlated with adenofibromatous versus cystic OCCC (p = 0.266), although there was a trend toward the association of loss of ARID1A expression with cystic lesions among OCCCs with a substantial adenofibromatous component (≥10%) (Table 4). Similarly, there was no significant difference between ARID1A expression and structural patterns including papillary, tubulocystic, and solid features (p = 0.957) or between ARID1A immunoreactivity and nuclear grade (p = 0.232). Kaplan-Meier analyses were performed to determine if there was a correlation between ARID1A expression and clinical outcome. No significant difference in survival was found between ARID1A positive and negative cases (*P* = 0.97) (Figure 2).

### 2.2. Discussion

Genome wide mutational analysis has identified *ARID1A* as the most commonly mutated gene in OCCC. Inactivation mutations of *ARID1A* resulted in loss of expression in tumors and loss of tumor suppressor function of ARID1A. In this report, we asked if loss of ARID1A expression correlated with morphological features or had an influence on disease aggressiveness and treatment response in OCCC. Although a recent study of 132 OCCCs demonstrated a lack of significant correlation between absence of ARID1A expression and overall survival in OCCC patients [2], the current study represented a detailed correlation study of loss of ARID1A expression and a variety of clinicopathological features which have not yet been studied. Our results have several implications regarding the potential role of *ARID1A* mutations in the pathogenesis of OCCC.

The observation that 59% of OCCCs exhibited an ARID1A intensity score of 0 is similar to the frequency (57%) of ARID1A mutation among OCCCs in a previous report that used purified OCCC samples for mutational analysis [1]. The percentage of cases exhibiting loss of ARID1A expression in the current cohort is slightly higher than the 42% reported in a previous cohort [2]. Nevertheless, both studies indicate that loss of ARID1A protein expression is common among OCCCs. One of the explanations for the lack of correlation of ARID1A expression and clinical stage and overall survival is that the loss of ARID1A expression occurs very early during tumor development of OCCC. Normally, ARID1A protein may act as a gatekeeper to prevent excessive cellular proliferation and suppress tumorigenesis. It has been shown, for example, that ARID1A is essential for normal cell cycle arrest [9] and the transforming potential of antisense expression of ARID1A has been demonstrated in NIH3T3 cells [10]. As well, ARID1A expression was lost in atypical endometriosis, which represents precursor lesions of OCCC [2]. Therefore, loss of ARID1A protein expression may not be as critical to tumor progression as to tumor initiation, which would explain why there was no difference in terms of clinical stage and treatment outcome between cases with and without ARID1A expression.

One may ask whether pathogenesis differs between OCCCs with and without ARID1A expression. It has been proposed, based on a recent comprehensive clinicopathological study, that OCCC develops from two distinct morphological pathways [11]. One pathway derives from a cyst and the other from an adenofibroma. Classification of OCCCs into cystic and adenofibromatous groups reveals differences in several clinicopathological features associated with each morphological group, including frequency in association with endometriosis, stage distribution, histologic patterns, and clinical outcome. Although there was a trend of loss of ARID1A expression and cystic OCCC, the correlation was not significant. Similarly, based on the *ARID1A* mutational status in 38 previously analyzed OCCCs [1], we found there was no significant correlation between *ARID1A* mutations and either cystic or adenofibromatous features (p = 0.438; Fisher Exact test). Further studies should focus on determining the molecular difference between OCCCs with and without ARID1A expression using genome-wide approaches. Finally, those OCCCs with ARID1A expression may utilize other mechanisms such as genetic deletion or loss of expression of the ARID1A binding partner, BRG1, to inactivate the ARID1A-BRG1 chromatin remodeling complex.

## 3. Experimental Section

### 3.1. Tissue Materials

Formalin-fixed and paraffin-embedded OCCC tissues were obtained from the Department of Pathology at the University of Tokyo Hospital (89 cases) and the Department of Pathology at the National Taiwan University Hospital (60 cases). All tumors were primary and were retrieved from the archives of each institution. Hematoxylin and eosin stained slides were reviewed to confirm the diagnosis using the most recent criteria of the World Health Organization. The tumor samples were arranged in tissue microarrays to facilitate immunohistochemistry. At least 2 cores (3 mm) were punched out for each case, and immunoreactivity of ARID1A was evaluated on those available cores. Tissue collection was in accordance with institutional guidelines.

Clinical information including demographics, age at diagnosis, clinical stage based on International Federation of Gynecology and Obstetrics (FIGO), and survival time after treatment were obtained for patients from whom records were available for review. Correlations of ARID1A expression with the following clinical variables were evaluated: age, stage of carcinoma (stage I/II *vs.* stage III/IV), and retroperitoneal lymph node metastasis. Specifically, clinical stage was assessable in 121 cases for which the staging procedures were complete; the remaining 28 cases were not included in analysis due to either incomplete surgical procedures or missing data. Results of retroperitoneal lymph node dissection were obtainable in 70 cases. Follow-up information included overall survival and cancer-related death. The follow-up period was calculated from the date of surgery to the date of death or last clinical evaluation. The mean follow-up interval was 59.9 months (range 1–196 months). In this study, the cases with adenofibromatous features were defined as those non-cystic lesions containing any percentage of adenofibromatous component; cystic cases were defined as those with a grossly identifiable cyst. The distinction among different structural patterns (tubulo-cystic, solid, and papillary) was based on the predominant component in a given specimen. Nuclear grades were made according to their relative nuclear atypia and were classified as mild, moderate, or severe atypia.

### 3.2. Immunohistochemistry

Tissue sections (4 μm) were prepared from the tissue microarray blocks. In addition, whole sections were used for analysis of tumors in the correlation study of *ARID1A* mutation and expression. Antigen retrieval was performed on deparaffinized sections by autoclaving them at 120 °C in a citrate buffer (pH 6.0) for 5 minutes. Slides were placed onto a Ventana Benchmark^®^ XT autostainer (Ventana Medical Systems Inc, Tucson, AZ) and immunohistochemical reactions were performed according to standard techniques of the autostainer. A commercially available monoclonal anti-ARID1A antibody, clone 3H2 (Sigma-Aldrich), was used at an optimal dilution of 1:25.

Because the distribution of ARID1A immunoreactivity was almost always homogenous within a given tumor, we used intensity scores of 0 (undetectable), 1+ (weakly positive), or 2+ (strong positive) to evaluate ARID1A immunoreactivity. A positive reaction was defined as discrete localization of the chromogen in the nuclei. The tissues were scored in a blinded fashion without knowledge of clinical information. Because we were focused on the loss of expression, we divided the cases into two groups: those scored as 1+ or 2+, and those scored as 0. To correlate the *ARID1A* mutational status and immunoreactivity, we selected 12 clear cell carcinomas for which the ARID1A mutational status was known from our previous study [1].

### 3.3. Statistical Analysis

Statistical analysis was performed using the Fisher’s exact test. Overall survival of OCCC cases was calculated using the Kaplan-Meier method, and statistical analyses were performed using the log-rank test. Statistical analyses were performed with StatView 5.0 software (SAS Institute, Cary, NC, USA) and *P* < 0.05 was considered statistically significant.

## 4. Conclusion

In conclusion, results from this study provided cogent evidence that ARID1A immunoreactivity did not correlate with any clinicopathological features examined in OCCC. It is likely that loss of ARID1A protein expression is not as important for tumor progression or response to treatment as it is for tumor initiation. Future studies will be required to define the molecular mechanisms by which loss of ARID1A expression contributes to tumor development in OCCC.

## Figures and Tables

**Table 1.** ARID1A expression in ovarian clear cell carcinoma.

**Immunostaining intensity score**	**Number of cases**	
0	88	Negative, n = 88
1+	36	] Positive, n = 61
2+	25	

Total	149	

**Table 2.** ARID1A expression in adnexal endometriosis and endometrial glands.

**Staining intensity score**	**Adnexal endometriosis**	**Endometrium Proliferative**	**Endometrium Secretory**	**Endometrium Menstrual**	**Endometrium Gestational**
0	0	0	0	0	0
1+	3	0	2	2	6
2+	2	11	9	2	6

Total	5	11	11	4	12

**Table 3.** Correlation of ARID1A immunoreactivity and mutation status in ovarian clear cell carcinoma.

	**Mutant**	**Wild-type**
IHC+	0	2
IHC−	9	1

p = 0.0073

**Table 4.**
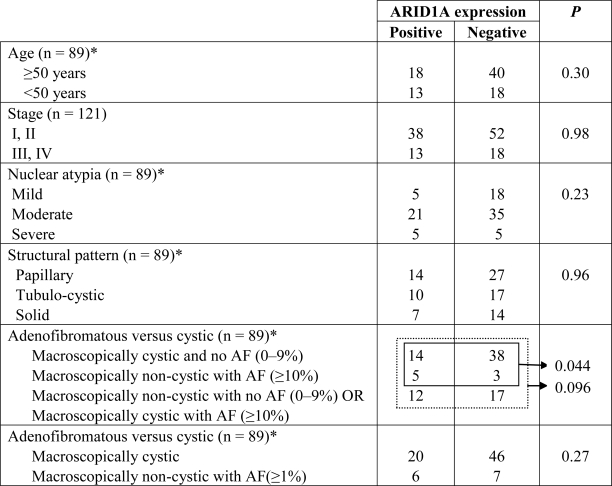
Correlation of ARID1A expression with clinicopathological features of ovarian clear cell carcinomas.
